# Supplementary material for: USP32 regulates late endosomal transport and recycling through deubiquitylation of Rab7
Source: Nat Commun. 2019 Mar 29;10:1454. doi: 10.1038/s41467-019-09437-x (PMC6440979; doi:10.1038/s41467-019-09437-x)
Supplement: Supplementary file 1 — Supplementary Information [file 41467_2019_9437_MOESM1_ESM.pdf]

Supplementary Information

USP32 regulates late endosomal transport and recycling through deubiquitylation of Rab7

Sapmaz and Berlin et.al.

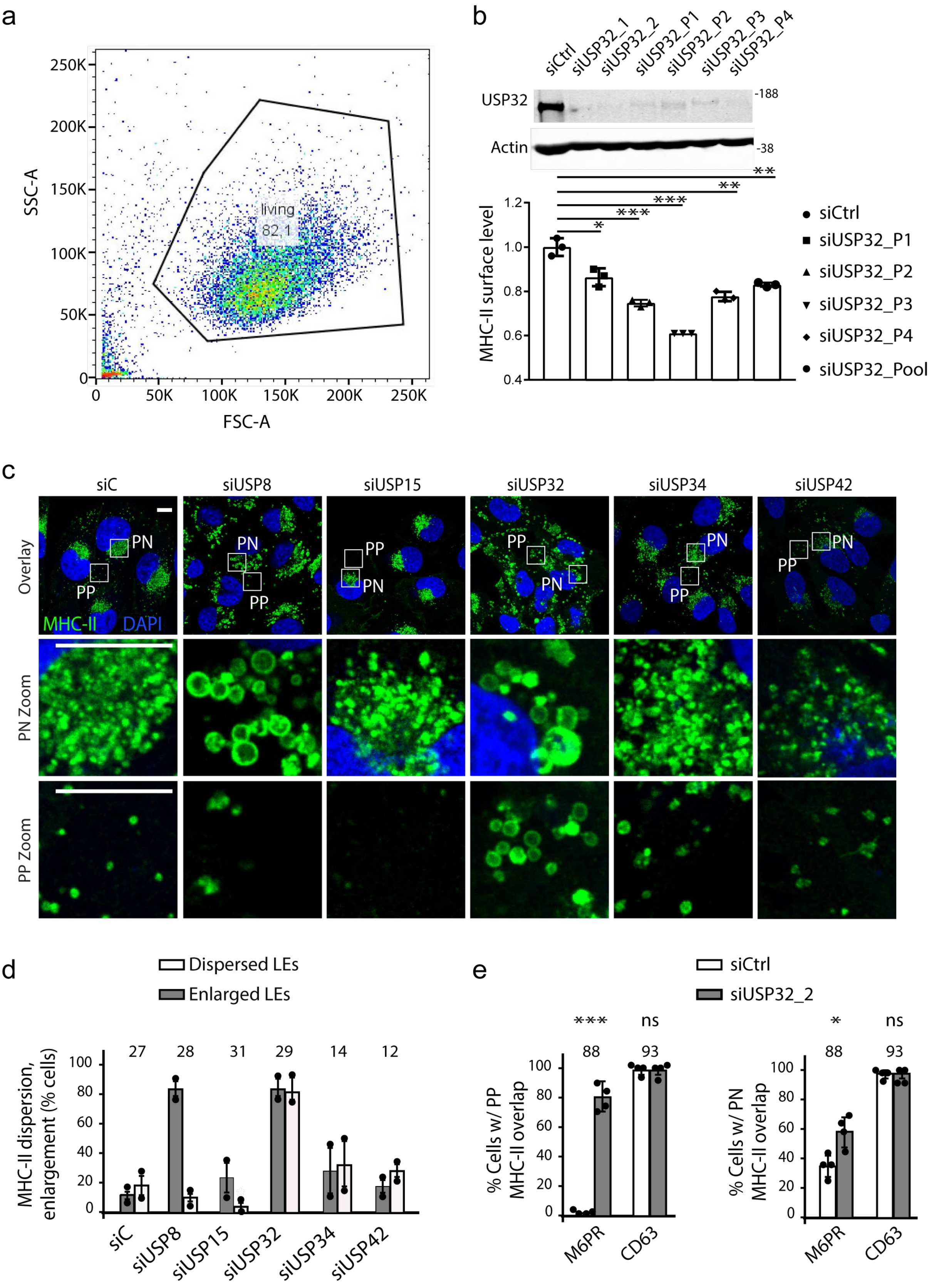

Supplementary Figure 1. Depletion of USP32 affects MHC class II surface levels and disturbs the architecture of the late endosomal compartment. (a) FACS gating strategy

employed in the siRNA DUB screen to select living MelJuSo cells. Mean fluorescent intensity (MFI) was determined by calculating the geometric mean. SSC-A: cell side scatter parameter, FSC-A: cell forward scatter. Related to Fig. 1a. (b) Validation of the effects of USP32 depletion on the steady-state MHC class II (MHC-II) surface levels. MelJuSo cells transfected with individual oligos from the siGENOME pool (P1-P4) or previously published custom oligos targeting USP32 (1 and 2) versus non-targeting control (siCtrl) were analyzed for surface expression of peptide-loaded MHC-II (expressed relative to control) by flow cytometry using monoclonal antibodies (L243-Cy3), n=3 independent experiments. Effects of individual siRNAs targeting USP32 on endogenous USP32 protein levels were validated by immunoblot of the corresponding cell lysates. Related to Fig. 1a. (c) Representative confocal images of MelJuSo cells transfected with siRNAs targeting the indicated DUBs, including USP8 (positive control) and USP32. Cells were fixed and stained for MHC-II (green) and nuclear DAPI (blue). Boxed regions highlight select perinuclear (PN) and peripheral (PP) regions. Scale bars = 10 μm. (d) % of cell population exhibiting MHC-II-positive late endosome (LE) dispersion (white bars) and/or enlargement (gray bars). Bar graphs report mean of independent experiments (black circles, n=2). Related to Fig. 1c. (e) % cells harboring enlarged MHC-II-positive vesicles co-labeled with the LE marker CD63 or TGN cargo mannose-6-phosphate receptor (M6PR) in either the cell periphery (PP, left graph) or perinuclear region (PN, right graph). Bar graphs report mean of independent samples (black circles, n=4). Related to Fig. 1d. Number of cells analyzed per condition appear above each bar. All error bars correspond to +/- s.d., significance calculated using Student's t-test: * $p<0.05$, ** $p<0.01$, *** $p<0.001$, ns = not significant.

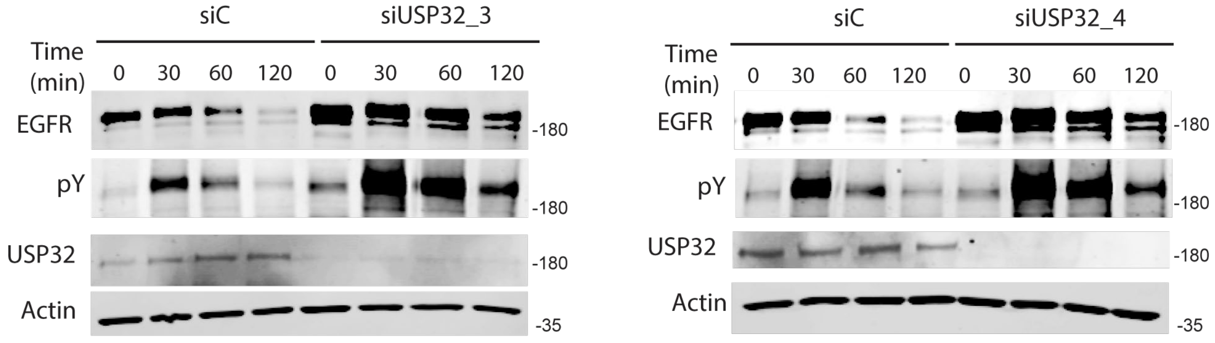

Supplementary Figure 2. Depletion of USP32 inhibits ligand-mediated turnover of EGFR. Effects of USP32 depletion using 2 independent siRNAs siUSP32_3 and siUSP32_4 (corresponding to oligos #P1 and #P4 from the siGENOME pool, respectively) on ligand-mediated degradation of EGFR. HeLa cells transfected as indicated were serum starved and incubated with EGF (25 ng/ml) for 0, 30, 60, or 120 min. Immunoblots against total (EGFR) and phosphorylated (pY) EGFR are shown, with actin as a loading control. Related to Fig. 2g.

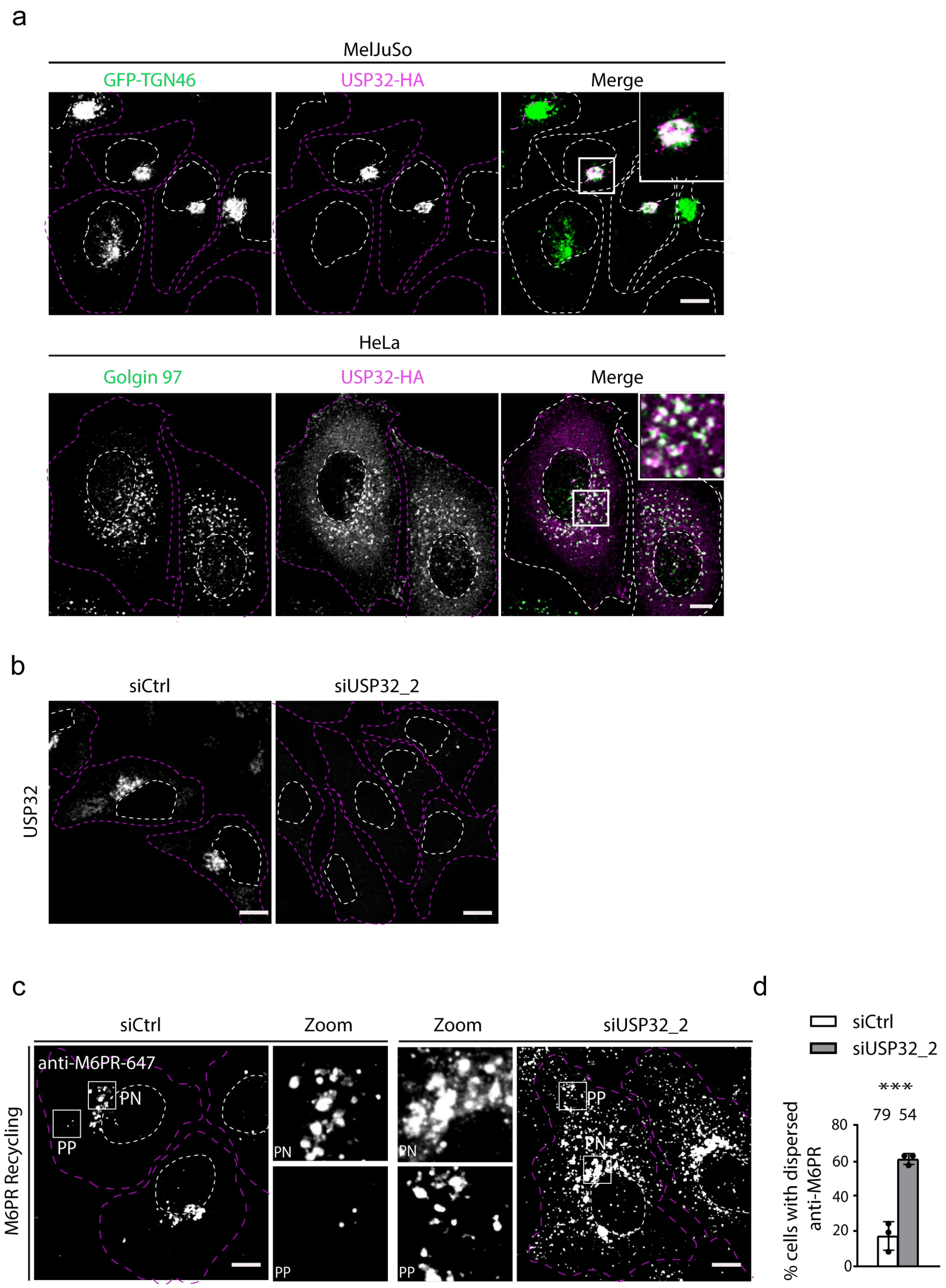

Supplementary Figure 3. USP32 partially colocalizes with the TGN and affects trafficking of M6PR.

(a) Localization of ectopically expressed USP32 to the TGN. Representative confocal images of ectopically expressed USP32-HA (magenta) in either MelJuSo cells stably expressing TGN46-GFP (green, top panels) or HeLa cells immunostained against the TGN marker Golgin-97 (green, bottom panels). Related to Fig 3a. (b) Representative confocal images of endogenous USP32 (white) in MelJuSo cells transfected as indicated and immunostained against endogenous USP32. (c) Effect of USP32 depletion on M6PR trafficking. Representative confocal z-projections (3D) of HeLa cells transfected as indicated and incubated with the mouse anti-CI-M6PR antibody (1 µg/ml, white) for 60 min, fixed and stained with anti-mouse-Alexa647 for visualization are shown with perinuclear (PN) and peripheral (PP) zooms. (d) % cells harboring dispersed anti-CI-M6PR antibody. Bars report mean of independent samples (black circles, n=3). Cell and nuclear boundaries are demarcated with dashed lines. All scale bars = 10µm. Total number of cells analyzed per condition appear above each bar. Error bars correspond to +/- s.d., with significance calculated using Student's t-test: *** $p<0.001$.

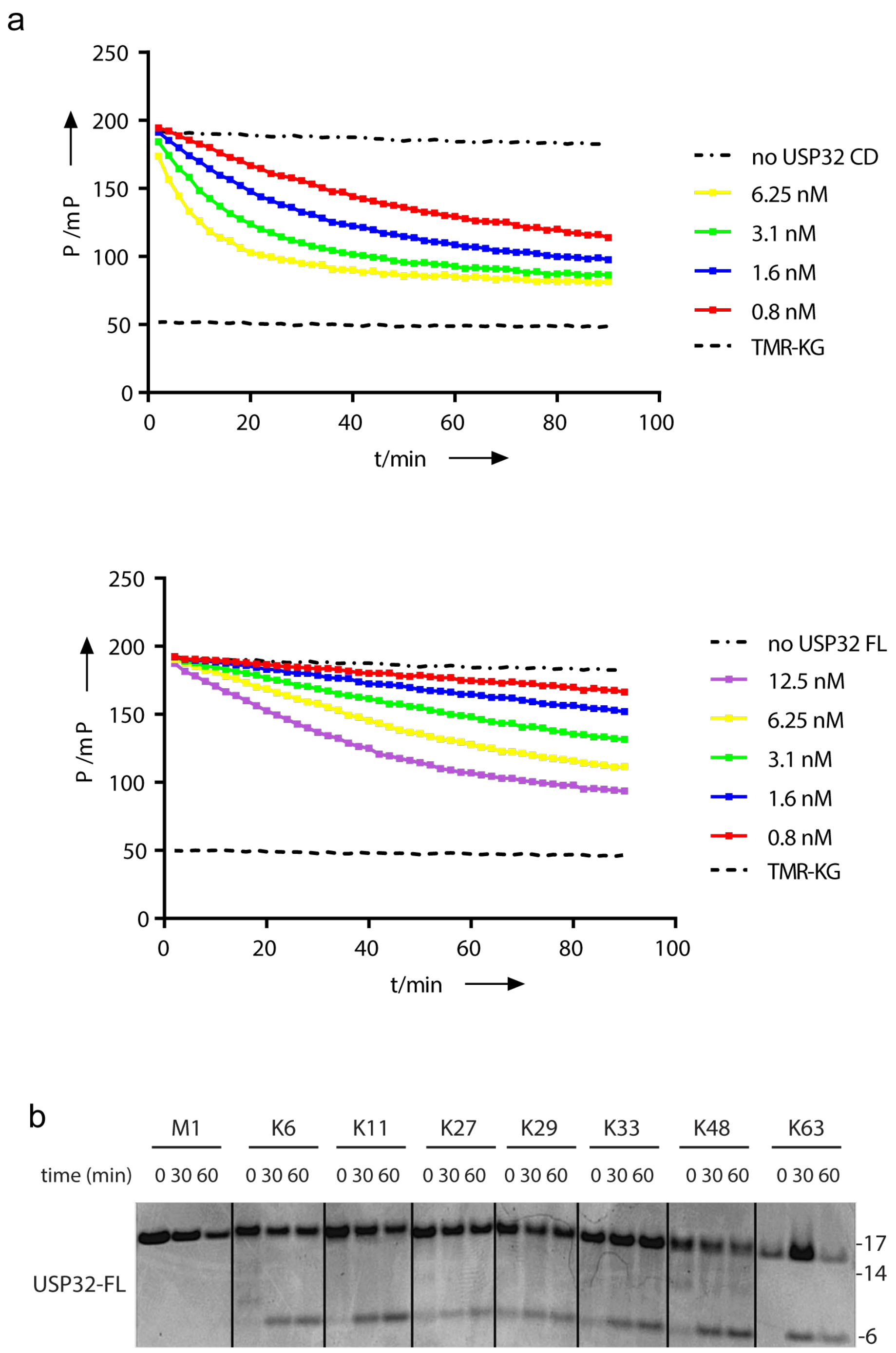

Supplementary Figure 4. In vitro characterization of USP32 catalytic activity. (a) Ub-FP activity assay for full length USP32 (FL) and its catalytic domain (CD) using different enzyme concentrations with 100 nM TMR-K(Ub)G (TMR-KG) substrate. Fluorescence data were recorded at 2 min intervals for 1.5 h. TMR-KG was used as a tracer to obtain lowest polarization value. P: polarization, y-axis defines polarization value in milliP (mP). (b) Di-ubiquitin cleavage assay of USP32 FL performed using eight ubiquitin linkage types (M1, K6, K11, K27, K29, K33, K48 and K63). Related to Fig. 3b.

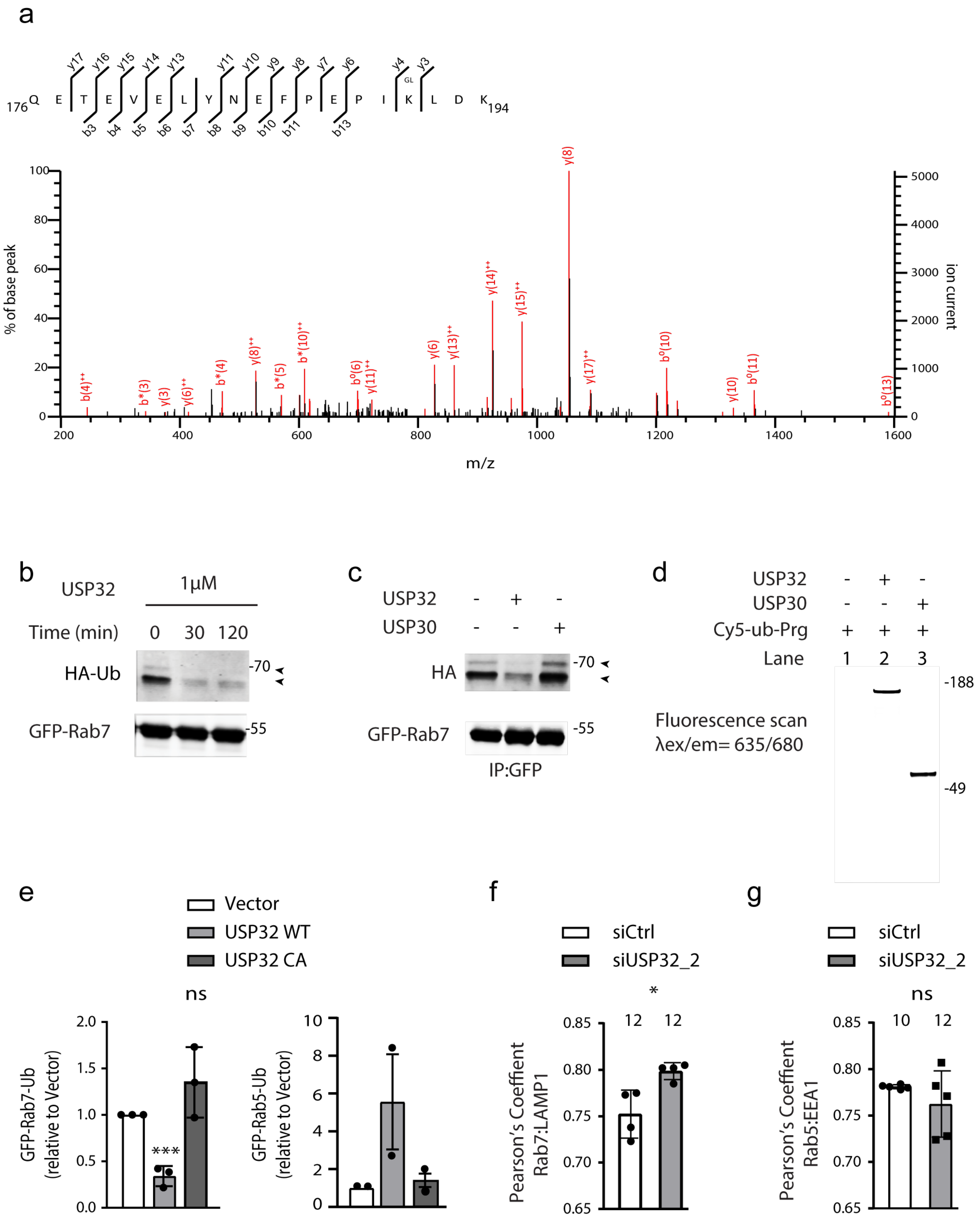

Supplementary Figure 5. Identification and validation of Rab7 as a substrate of USP32. (a) Identification of Rab7 ubiquitination on K191 using mass spectrometry. MelJuSo cells were transfected with either control siRNA or one targeting USP32 (siUSP32_2), and affinity-enriched ubiquitinated material was analyzed by tandem mass spectrometry (MS/MS). The

MS/MS spectrum shown derives from a tryptic peptide 176-194 matching Rab7 (Uniprot accession nr P55149). Fragment ions of the b- and y-ion series are indicated, and the GlyGly modification maps to K191. Related to Fig 4b. (b, c) GFP-Rab7, immunoprecipitated (IP) from HEK293T cells ectopically coexpressing HA-tagged ubiquitin (HA-Ub), was incubated with 1 μM final concentration of purified (b) USP32 enzyme over time (0, 30, 120 min) or (c) USP32 versus USP30 as indicated for 30 min, and remaining ubiquitination of GFP-Rab7 was assessed by immunoblot against HA. Arrows mark ubiquitylated GFP-Rab7 species. (d) Purified USP32 or USP30 (catalytic domain) were reacted with the Cy5-Ub-Prg probe, separated by 4-12% SDS-PAGE and analyzed by Typhoon FLA 9500 Image System using 635 nM excitation and 680 nM emission filters. (e) Quantification of GFP-Rab7 (n=3 independent experiments) and GFP-Rab5 (n=2 independent experiments) ubiquitination as a function of USP32 activity (relative to GFP) normalized to vector control (lane 2). Related to Fig. 4d. (f, g) Colocalization of (f) Rab7 with late endosome marker LAMP1 or (g) Rab5 with early endosome marker EEA1 expressed as Pearson's coefficients. Bars report mean of values calculated from multicell images (black circles) from n=2 independent experiments. Number of cells analyzed per condition appear above each bar. Related to Fig. 5a. Error bars correspond to +/- s.d., with significance calculated using Student's t-test: * p<0.05, *** p<0.001, ns = not significant.

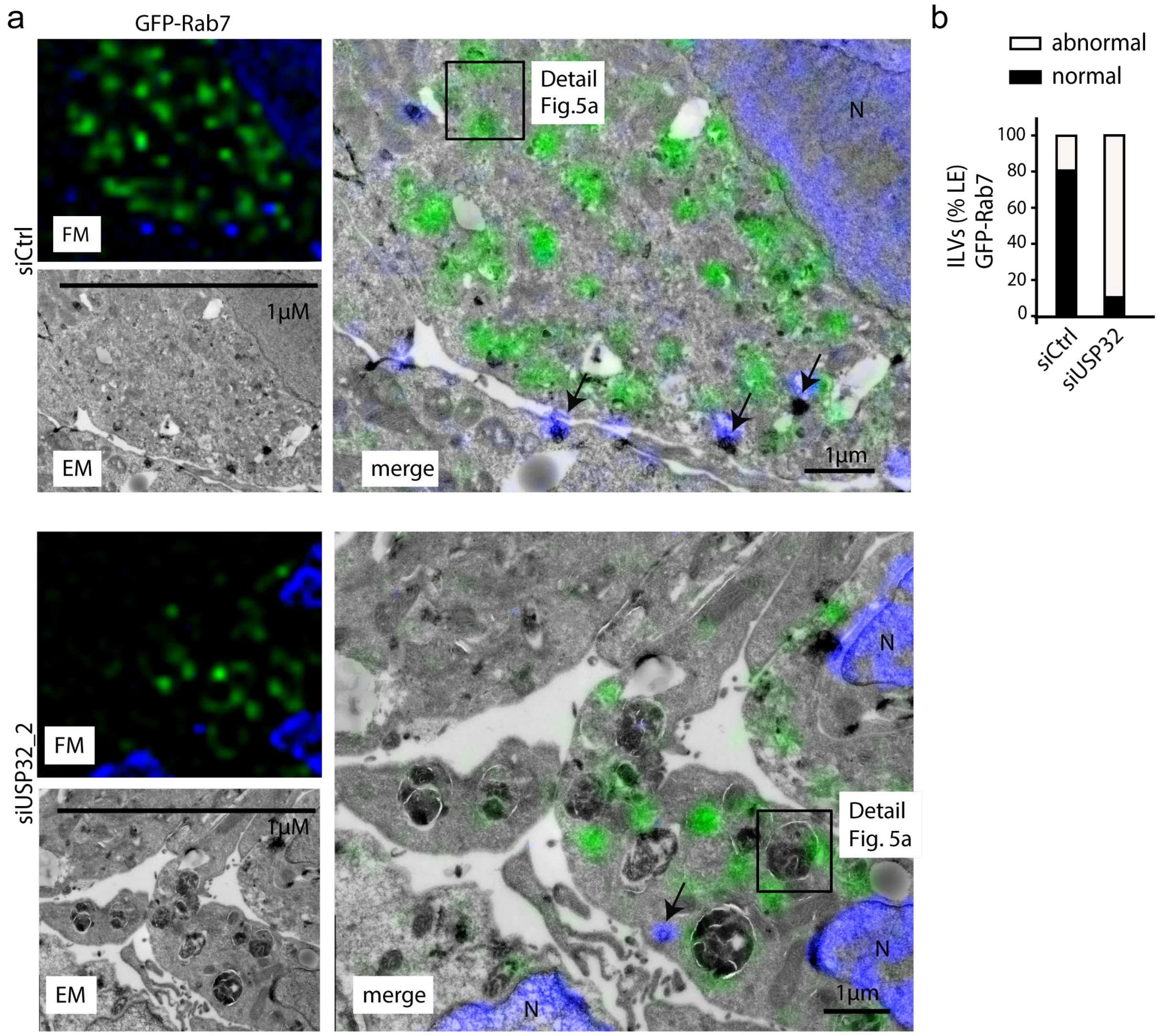

Supplementary Figure 6. Ultrastructural characterization of late endosomal defects arising from USP32 depletion in cells expressing GFP-Rab7. (a) CLEM of late endosomes (LEs) in control (siCtrl, top panels) versus USP32-depleted (siUSP32_2, bottom panels) MelJuSo cells stably expressing GFP-Rab7. N: DAPI-stained nucleus, arrow: fluorescent microspheres. (b) Quantification of intraluminal vesicles (ILVs) of LEs exhibiting normal (black) or abnormal (white) contents in cells treated with either siCtrl or siUSP32_2. All scale bars = 1µm. Related to Fig. 5c, d.

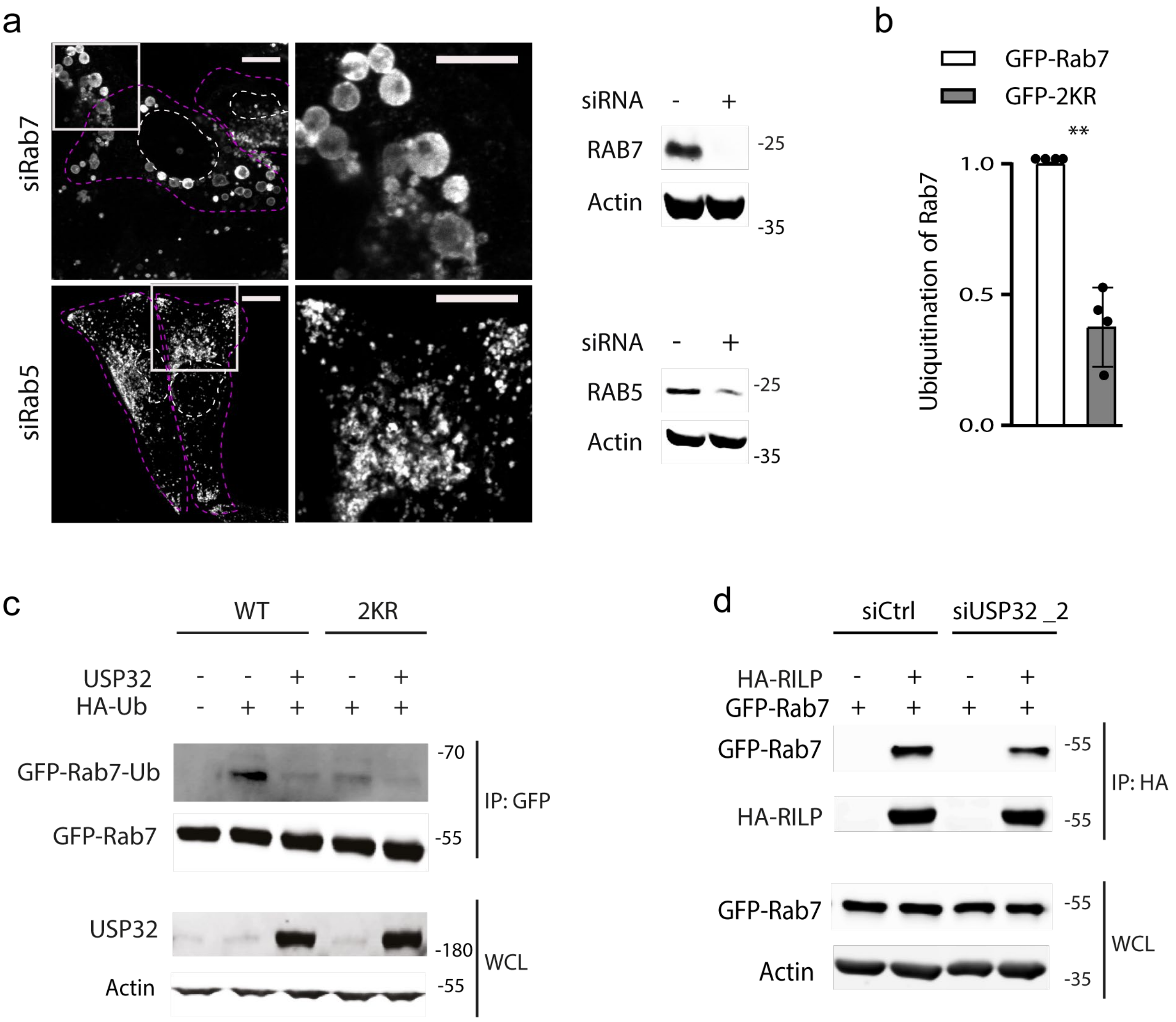

Supplementary Figure 7. Effects of Rab7 versus Rab5 on late endosomal architecture and characterization of Rab7 mutant K191R, K194R (2KR). (a) Effects of Rab7 and Rab5 depletion on the size and intracellular distribution of the MHC class II compartment. Representative confocal images of MHC-II (white) along with the corresponding immunoblot analyses of cells transiently transfected with siRNAs targeting expression of the indicated proteins (+) compared to siCtrl (-) are shown. Cell and nuclear boundaries are demarcated with dashed magenta and white lines, respectively. Related to Fig. 5e. (b) Quantification of steady-state ubiquitylation on GFP-Rab7 versus its mutant 2KR. Bars report mean of n=4 independent experiments (black circles). Error bars correspond to +/- s.d., with significance calculated using Student's t-test, ** p<0.01. (c) Ubiquitylation status analysis of GFP-Rab7-WT versus GFP-Rab7-2KR as a function of USP32 catalytic activity. GFP-Rab7 or 2KR was immunoprecipitated from HEK293T cells coexpressing HA-Ub and either USP32 WT or neither, followed by immunoblot against HA and GFP; WCL: whole cell lysate. (d) Co-

immunoprecipitation (Co-IP) of HA-RILP with GFP-Rab7 from HeLa cells transfected with siCtrl or siUSP32 as indicated. Related to Fig. 6f.

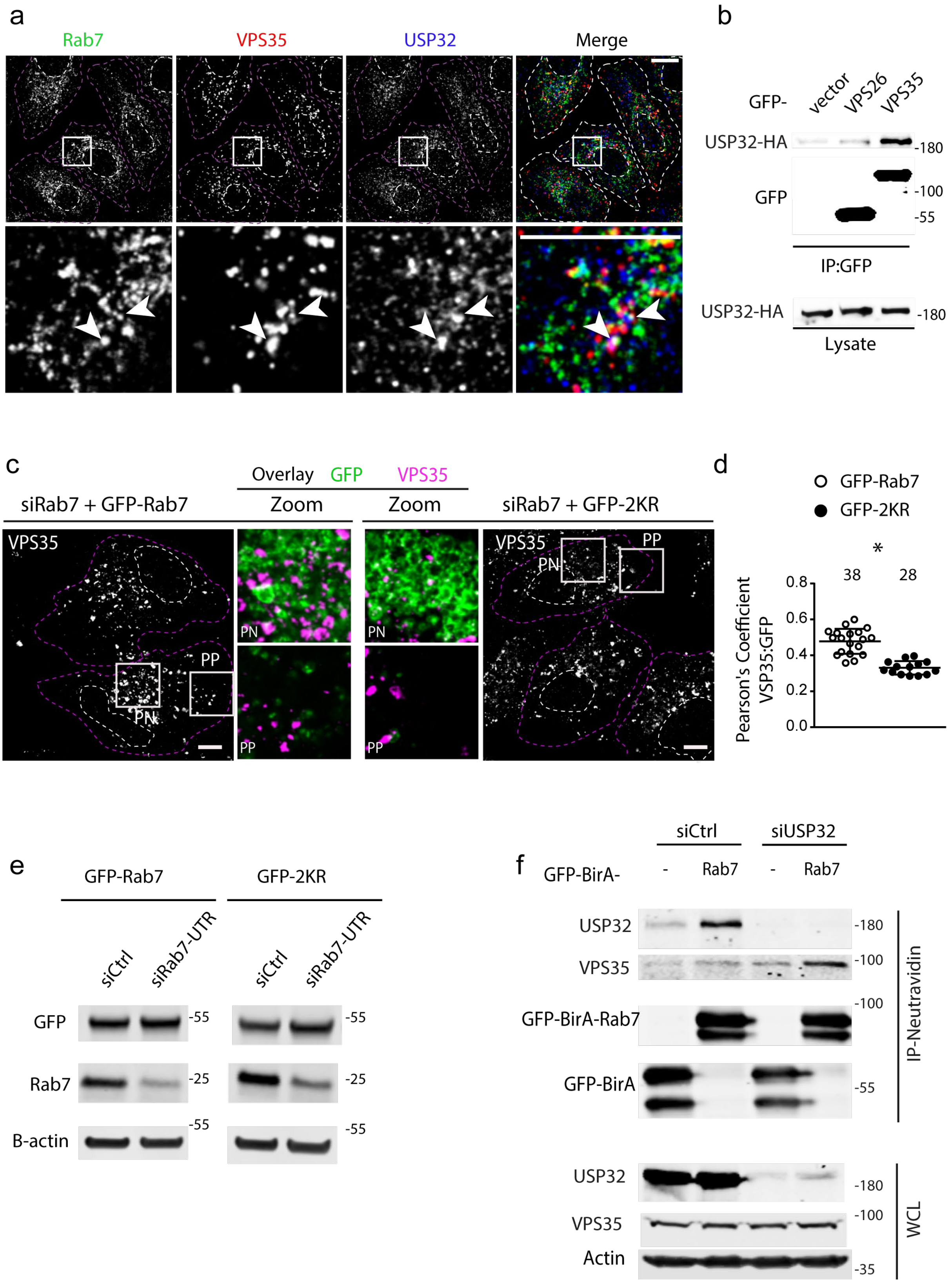

Supplementary Figure 8. USP32 interacts and colocalizes with VPS35 and Rab7 on vesicular structures. (a) Representative confocal images of HeLa cells immunostained against endogenous Rab7 (green), VPS35 (red), and USP32 (blue) are shown. Cell and nuclear boundaries are demarcated with dashed lines. Arrows highlight instances of juxtaposition or colocalization between all 3 channels. Boxed regions highlight zoom-ins. (b) Co-immunoprecipitation (Co-IP) of HA-USP32 with retromer complex components RFP-VPS26 and RFP-VPS35 from HEK293T cells transfected as indicated. (c) Representative confocal images of MelJuSo cells transferred with the indicated siRNAs and labeled for VPS35 (white) are shown. Boxed perinuclear (PN) and peripheral (PP) region overlays of VPS35 (magenta) with GFP-Rab7 or GFP-2KR (green) are shown. (d) Colocalization between VPS35 and GFP-Rab7 (open circles) or GFP-2KR (closed circles) expressed as Pearson's coefficient calculated from multicell images. Number of cells analyzed per condition appear above each scatter, n=2 independent experiments. Error bars correspond to +/- s.d., with significance calculated using Student's t-test, * $p<0.05$. (e) Silencing of endogenous Rab7 in MelJuSo cells stably expressing either GFP-Rab7 or GFP-2KR using siRNA targeting the UTR of Rab7 (siRab7-UTR). Immunoblots against Rab7, GFP and actin are shown. (f) Proximity-based labeling of VPS35 with biotin by GFP-BirA-Rab7 as a function of USP32 depletion (siUSP32) using oligo 2. Related to Fig. 7h.

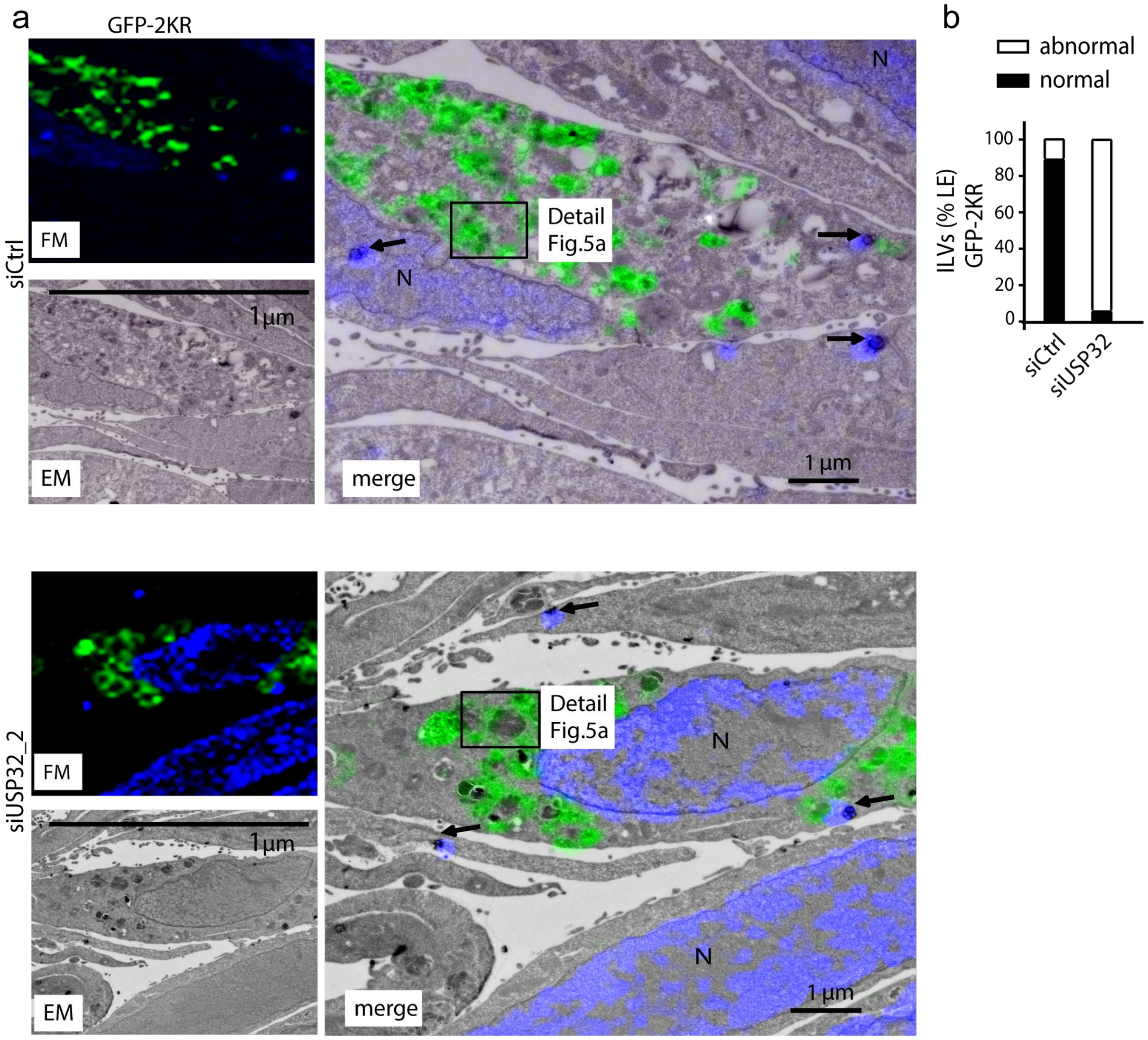

Supplementary Figure 9. Ultrastructural characterization of late endosomal defects arising from USP32 depletion in cells expressing GFP-Rab7-2KR. (a) CLEM of LEs in control (siCtrl, top panels) versus USP32-depleted (siUSP32_2, bottom panels) MelJuSo cells stably expressing GFP-2KR. N: DAPI-stained nucleus, arrow: fluorescent microspheres. (b) Quantification of intraluminal vesicles (ILVs) exhibiting normal (black) or abnormal (white) contents in cells treated with either siCtrl or siUSP32_2. All scale bar = 1 µm. Related to Fig. 8c, d.

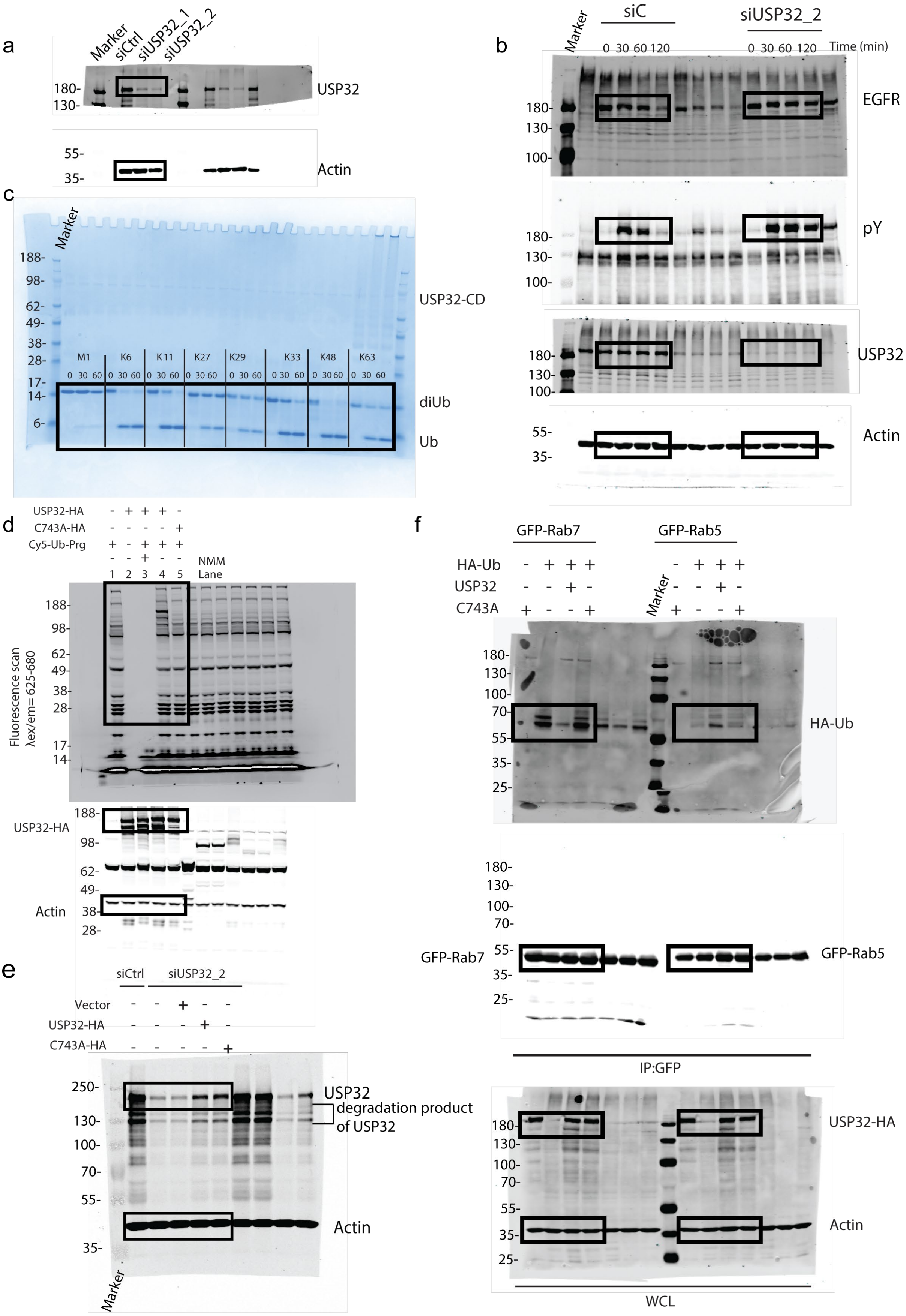

a
Marker
siCtrl
siUSP32_1
siUSP32_2
180-
130-
USP32
55-
35-
Actin
b
Marker
siC
siUSP32_2
0 30 60 120
0 30 60 120
Time (min)
180-
130-
100-
EGFR
pY
USP32
55-
35-
Actin
c
Marker
188-
98-
62-
49-
38-
28-
17-
14-
6-
M1
K6
K11
K27
K29
K33
K48
K63
0 30 60
USP32-CD
diUb
Ub
d
USP32-HA
C743A-HA
Cy5-Ub-Prg
NMM
Lane
1 2 3 4 5
Fluorescence scan
λex/em= 625-680
188-
98-
62-
49-
38-
28-
17-
14-
USP32-HA
Actin
e
siCtrl
siUSP32_2
Vector
USP32-HA
C743A-HA
250-
130-
100-
70-
55-
35-
USP32
degradation product
of USP32
Actin
Marker
f
GFP-Rab7
GFP-Rab5
HA-Ub
USP32
C743A
Marker
180-
130-
100-
70-
55-
35-
25-
HA-Ub
GFP-Rab7
GFP-Rab5
IP:GFP
USP32-HA
Actin
WCL

Supplementary Figure 10. Uncropped and unprocessed gel images presented in Figs. 1-4. (a) Related to Fig.1c: USP32 protein expression in response to depletion of USP32 with two independent siRNA oligos (1 and 2, top panel) with actin as loading control (bottom panel). (b) Related to Fig. 2f: total EGFR (top panel) and phosphorylated (pY) EGFR (second from top panel) together with USP32 (second from bottom panel) expression levels with actin (bottom panel) as loading control. (c) Related to Fig. 3b: in vitro cleavage of diubiquitin linkages (M1, K6, K11, K27, K29, K33, K48 and K63) by the catalytic domain (CD) of USP32 (USP32-CD). (d) Related to Fig. 3d: in-gel fluorescence scanning (top panel) and immunoblot (bottom panel) of DUB activity-based probe assay performed on lysates of HEK293T cells expressing USP32-HA or catalytic mutant C743A-HA in the absence (-) or presence (+) of Cy5-Ub-Prg probe. (e) Related to Fig. 3e: anti-HA and anti-actin immunoblots corresponding to rescue of USP32 depletion (siUSP32_2) by siRNA-resistant USP32-HA or catalytic mutant C743A-HA. (f) Related to Fig. 4d: ubiquitylation status of GFP-Rab7 versus GFP-Rab5 as a function of USP32 catalytic activity. Blackline square on each full-length gel or blot demarcates cropping for presentation in the associated main figures.

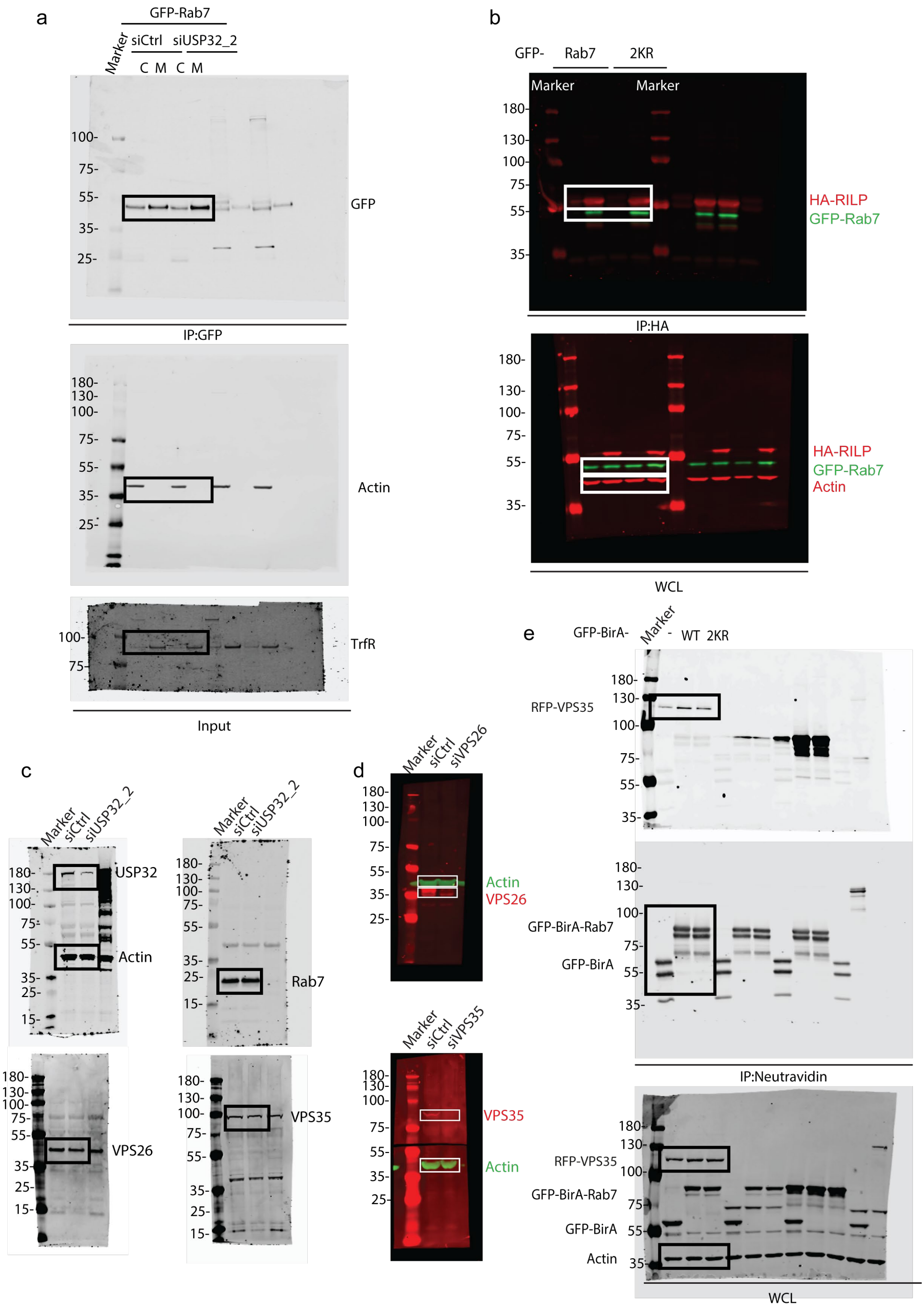
a
GFP-Rab7
Marker siCtrl siUSP32_2
C M C M
100-
75-
55-
35-
25-
GFP
IP:GFP
180-
130-
100-
75-
55-
35-
25-
Actin
100-
75-
TrfR
Input
b
GFP- Rab7 2KR
Marker Marker
180-
130-
100-
75-
55-
35-
HA-RILP
GFP-Rab7
IP:HA
180-
130-
100-
75-
55-
35-
HA-RILP
GFP-Rab7
Actin
WCL
c
Marker siCtrl siUSP32_2
180-
130-
100-
75-
55-
35-
25-
15-
USP32
Actin
Marker siCtrl siUSP32_2
180-
130-
100-
75-
55-
35-
25-
15-
Rab7
180-
130-
100-
75-
55-
35-
25-
15-
VPS26
180-
130-
100-
75-
55-
35-
25-
15-
VPS35
d
Marker siCtrl siVPS26
180-
130-
100-
75-
55-
35-
25-
Actin
VPS26
Marker siCtrl siVPS35
180-
130-
100-
75-
55-
35-
25-
VPS35
Actin
e
GFP-BirA- Marker - WT 2KR
180-
130-
100-
75-
55-
35-
RFP-VPS35
180-
130-
100-
75-
55-
35-
GFP-BirA-Rab7
GFP-BirA
IP:Neutravidin
180-
130-
100-
75-
55-
35-
RFP-VPS35
GFP-BirA-Rab7
GFP-BirA
Actin
WCL

Supplementary Figure 11. Uncropped and unprocessed immunoblots corresponding to data presented in Figs. 5-7. (a) Related to Fig. 5g: membrane-bound versus cytosolic GFP-Rab7 (top panel) isolated by immunoprecipitation (IP) from corresponding fractions of lysates from MelJuSo cells stably expressing GFP-Rab7 and transfected as indicated. Actin (middle panel) and TrfR (bottom panel) were used as controls for the input levels of cytosolic and membrane fractions, respectively. (b) Related to Fig. 6d: co-immunoprecipication (IP, top panel) of GFP-Rab7 (green) with HA-RILP (red). Whole cell lysates (WCL, bottom panel) show expression levels of HA-RILP (red) and GFP-Rab7 (green) together with actin (red) as loading control. (c) Related to Figs. 5f and 7c: expression level of Rab7 (top right panel), VPS35 (bottom right panel) and VPS26 (bottom left level) in response to depletion of USP32 (top left panel) with siUSP32_2 oligo. Actin (top left panel) was used as loading control. (d) Related to Fig. 7a: depletion of VPS26 (top panel, red) and VPS35 (bottom panel, red) protein levels with siGENOME siRNAs. Actin (top and bottom panel, green) was used as loading control. (e) Related to Fig. 7a: IP of biotinylated RFP-VPS35 (top panel) with GFP-BirA-Rab7 versus GFP-BirA-2KR (middle panel) from HEK293T cells. Immunoblots of WCL for GFP, RFP and actin are shown in the bottom panel. Blackline or whiteline square on each full-length blot demarcates cropping for presentation in the associated main figures.

Supplementary Table 1. The list of primers used in this study

Primer name	Primer sequence 5'-3'
USP32_HA/GFP Forward	ACGCGTCGACGATGGGTGCCAAGGAGTCACGGATC
USP32_HA/GFP Reverse	CCGGGATCCCGCTGTAACACACAGTACTTTTTGTAATCAGACTC
USP32 C743A Forward	GCAATCTGGGAAACACAGCCTTCATGAACTCAAGC
USP32 C743A Reverse	CGTTAGACCCTTTGTGTCGGAAGTACTTGAGTTCG
USP32 rescue Forward	GATAGAATTGGAAGCAGCCTGAGCTATGTCAACACTACAGAAGAGAAATTTTC
USP32 rescue Reverse	GAAAATTTCTCTTCTGTAGTGTTGACATAGCTCAGGCTGCTTCCAATTCTATC
USP32FL-pFastBac Forward	CAGGGACCCGGTGGTGCCAAGGAGTCACGGATC
USP32CD-pFastBac Forward	CAGGGACCCGGTAGTAAAATAGATAGACACAAGGTTCCCACAG
USP32FL/CD-pFastBac Reverse	CGAGGAGAAGCCCGGTTACTGTAACACACAGTACTTTTTGTAATCAGACTC
Rab7 K191R/K194R Forward	GAATTCCCTGAACCCATCAGACTGGACAGGAACGACCGGGCCAAGG
Rab7 K191R/K194R Reverse	CCTTGGCCCGGTCGTTCCTGTCCAGTCTGATGGGTTCAGGGAATTC
VPS26_N1 Forward	ACGCGTCGACGATGAGTTTTCTTGGAGGCTTTTTTGGTCC
VPS26_N1 Reverse	CCGGGATCCCGCATTTCAGGCTGTTCGGCAGATGCC
VPS35_C1 Forward	GACGGTACCATGCCTACAACACAGCAG
VPS35_C1 Reverse	GGTGGATCCTTAAAGGATGAGACCTTCAT
Rab5_C1 Forward	CCCAGGATCCATGGCTAATCGAGGAGCAAC
Rab5_C1 Reverse	CCCAAAGCTTTTACTACAACACTGACTCCTGG
Mp mGFP-linker-BirA* Forward	GGCATGGACGAGCTGTACAAGGGCGCCAGCGGACCCAAGGACAACACCGTGCCC
NheI mGFP Forward	ACTACGGCTAGCACCACCATGGTGAGCAAG
BglII BirA* Reverse	ACTACGAGATCTCTTCTCTGCGCTTCTCAGGGAGA
USP32 sequencing 1	AACAGCTGAGTGATCTCTGTGG
USP32 sequencing 2	TTGTGGGGAAGACAGAGCTT
USP32 sequencing 3	TCTCGATGGCTTCTATCTGGA
USP32 sequencing 4	GACACCACAAAGATGGGTCA
USP32 sequencing 5	CTGAAGCCTCAGAAACTGCT
USP32 sequencing 6	CCCCGCTATCTTCTCTTCCT
USP32 sequencing 7	TGAACTCAACAGGACAAATCC
USP32 sequencing 8	CTAATAGCAGCCCACGGACT
