# Supplementary material for: USP32 regulates late endosomal transport and recycling through deubiquitylation of Rab7
Source: Nat Commun. 2019 Mar 29;10:1454. doi: 10.1038/s41467-019-09437-x (PMC6440979; doi:10.1038/s41467-019-09437-x)
Supplement: Supplementary file 3 — Description of Additional Supplementary Files [file 41467_2019_9437_MOESM3_ESM.pdf]

Description of Additional Supplementary Files

Supplementary Movie 1. Intracellular localization and dynamics of USP32. Time-lapse (5 sec per frame, 3 fps, scale bar = 10 µm) of MelJuSo cells ectopically expressing USP32-GFP (green) and labeled with Lysotracker DeepRed (magenta). Cell boundaries and nuclei are demarcated with dashed lines on the basis of t=0 sec. Related to Fig. 3a and Supplementary Fig. 3a, b.

Supplementary Movie 2. Zoom-in taken from Supplementary movie 1 highlighting transient interactions between USP32-GFP (green) and acidified organelles marked by Lysotracker DeepRed (magenta). Related to Fig. 3a, Supplementary Fig. 3a, b and Supplementary movie 1.

Supplementary Movie 3. Dynamics and interactions of the TGN with acidic compartments. Timelapse (5 sec per frame, 10 fps, scale bar = 10 µm) of control MelJuSo cells (siCtrl) stably expressing TGN46-GFP (green) and labeled with Lysotracker DeepRed (magenta). Cell boundaries and nuclei are demarcated with dashed lines on the basis of t=0 sec. Related to Fig. 3a and Supplementary Fig. 3a, b.

Supplementary Movie 4. Perinuclear zoom-in of Supplementary movie 3 highlighting transient interactions between TGN46-GFP (green) and acidified organelles marked by Lysotracker DeepRed (magenta). Related to Fig. 3a, Supplementary Fig. 3a, b and Supplementary movie 3.

Supplementary Movie 5. Effect of USP32 depletion on dynamics and interactions of the TGN with acidic compartments. Time-lapse (5 sec per frame, 10 fps, scale bar = 10 µm) of MelJuSo cells stably expressing TGN46-GFP (green) depleted of USP32 (siUSP32) and labeled with Lysotracker DeepRed (magenta). Cell boundaries and nuclei are demarcated with dashed lines on the basis of t=0 sec. Related to Fig. 3a and Supplementary Fig. 3a, b.

Supplementary Movie 6. Perinuclear zoom-in of Supplementary movie 5 highlighting accumulation of TGN46-GFP (green) on acidified organelles marked by Lysotracker DeepRed (magenta). Related to Fig. 3a, Supplementary Fig. 3a, b and Supplementary movie 5.

Supplementary Movie 7. Effect of USP8 depletion on dynamics and interactions of the TGN with acidic compartments. Time-lapse (5 sec per frame, 10 fps, scale bar = 10 µm) of MelJuSo cells stably expressing TGN46-GFP (green) depleted of USP8 (siUSP8) and labeled with Lysotracker DeepRed (magenta). Cell boundaries and nuclei are demarcated with dashed lines on the basis of t=0 sec. Related to Fig. 3a and Supplementary Fig. 3a, b.

Supplementary Movie 8. Perinuclear zoom-in of Supplementary movie 7 highlighting accumulation of TGN46-GFP (green) on acidified organelles marked by Lysotracker DeepRed (magenta). Related to Fig. 3a, Supplementary Fig. 3a, b and Supplementary movie 7.

Supplementary Movie 9. Membrane dynamics of GFP-Rab7-positive endosomes. Time-lapse (5 sec per frame, 10 fps, scale bar = 10 µm) of control MelJuSo cells (siCtrl) stably expressing GFP-Rab7 (green) and labeled with Lysotracker DeepRed (magenta). Cell boundaries and nuclei are demarcated with dashed lines on the basis of t=0 sec. Related to Fig. 6a-c.

Supplementary Movie 10. Effect of USP32 depletion on membrane dynamics of GFP-Rab7-positive endosomes. Time-lapse (5 sec per frame, 10 fps, scale bar = 10 μm) of MelJuSo cells stably expressing GFP-Rab7 (green) depleted of USP32 (siUSP32_2) and labeled with Lysotracker DeepRed (magenta). Cell boundaries and nuclei are demarcated with dashed lines on the basis of t=0 sec. Related to Fig. 6a-c.

Supplementary Movie 11. Membrane dynamics of GFP-Rab7-2KR-positive endosomes. Time-lapse (5 sec per frame, 10 fps, scale bar = 10 μm) of control MelJuSo cells (siCtrl) stably expressing GFPRab7-2KR (GFP-2KR) (green) and labeled with Lysotracker DeepRed (magenta). Cell boundaries and nuclei are demarcated with dashed lines on the basis of t=0 sec. Related to Fig. 6a-c.

Supplementary Movie 12. Effect of USP32 depletion on membrane dynamics of GFP-Rab7-2KRpositive endosomes. Time-lapse (5 sec per frame, 10 fps, scale bar = 10 μm) of MelJuSo cells stably expressing GFP-Rab7-2KR (GFP-2KR) (green) depleted of USP32 (siUSP32_2) and labeled with Lysotracker DeepRed (magenta). Cell boundaries and nuclei are demarcated by dashed lines on the basis of t=0 sec. Related to Fig. 6a-c.

Supplementary Movie 13. Tubulation from GFP-Rab7-positive late endosomes. Selected zoom from a time-lapse (5 sec per frame, 3 fps, scale bar = 10 μm) of control MelJuSo cells (siCtrl) stably expressing GFP-Rab7 (green) and labeled with Lysotracker DeepRed (magenta). Related to Fig. 8a.

Supplementary Movie 14. Effect of USP32 depletion on bud and tubule resolution from GFP-Rab7-positive late endosomes. Selected zoom from a time-lapse (5 sec per frame, 3 fps, scale bar = 10 μm) of MelJuSo cells stably expressing GFP-Rab7 (green) depleted of USP32 (siUSP32_2) and labeled with Lysotracker DeepRed (magenta). Related to Fig. 8a.

Supplementary Movie 15. Tubulation from GFP-Rab7-positive late endosomes. Selected zoom from a time-lapse (5 sec per frame, 3 fps, scale bar = 10 μm) of control MelJuSo cells (siCtrl) stably expressing GFP-Rab7-2KR (GFP-2KR) (green) and labeled with Lysotracker DeepRed (magenta). Related to Fig. 8b.

Supplementary Movie 16. Effect of USP32 depletion on bud and tubule resolution from GFP-Rab7-positive late endosomes. Selected zoom from a time-lapse (5 sec per frame, 3 fps, scale bar = 10 μm) of MelJuSo cells stably expressing GFP-Rab7-2KR (GFP-2KR) (green) depleted of USP32 (siUSP32_2) and labeled with Lysotracker DeepRed (magenta). Related to Fig. 8b.
