# Supplementary material for: USP32 regulates late endosomal transport and recycling through deubiquitylation of Rab7
Source: Nat Commun. 2019 Mar 29;10:1454. doi: 10.1038/s41467-019-09437-x (PMC6440979; doi:10.1038/s41467-019-09437-x)
Supplement: Supplementary file 20 — Reporting Summary [file 41467_2019_9437_MOESM20_ESM.pdf]

nature research

Corresponding author(s): Huib Ovaa, Jacques Neefjes

Last updated by author(s): Feb 11, 2019

Reporting Summary

Nature Research wishes to improve the reproducibility of the work that we publish. This form provides structure for consistency and transparency in reporting. For further information on Nature Research policies, see Authors & Referees and the Editorial Policy Checklist.

Statistics

For all statistical analyses, confirm that the following items are present in the figure legend, table legend, main text, or Methods section.

n/a	Confirmed	
☐	☒	The exact sample size (*n*) for each experimental group/condition, given as a discrete number and unit of measurement
☐	☒	A statement on whether measurements were taken from distinct samples or whether the same sample was measured repeatedly
☐	☒	The statistical test(s) used AND whether they are one- or two-sided<br>*Only common tests should be described solely by name; describe more complex techniques in the Methods section.*
☐	☒	A description of all covariates tested
☐	☒	A description of any assumptions or corrections, such as tests of normality and adjustment for multiple comparisons
☐	☒	A full description of the statistical parameters including central tendency (e.g. means) or other basic estimates (e.g. regression coefficient) AND variation (e.g. standard deviation) or associated estimates of uncertainty (e.g. confidence intervals)
☐	☒	For null hypothesis testing, the test statistic (e.g. *F*, *t*, *r*) with confidence intervals, effect sizes, degrees of freedom and *P* value noted<br>*Give P values as exact values whenever suitable.*
☐	☒	For Bayesian analysis, information on the choice of priors and Markov chain Monte Carlo settings
☐	☒	For hierarchical and complex designs, identification of the appropriate level for tests and full reporting of outcomes
☐	☒	Estimates of effect sizes (e.g. Cohen's *d*, Pearson's *r*), indicating how they were calculated

Our web collection on statistics for biologists contains articles on many of the points above.

Software and code

Policy information about availability of computer code

Data collection	Flow cytometry data was collected using BD FACS DIVA (V8.0.1) software<br>Confocal microscopy data was collected using LAS-AF or LAS X software<br>Western blot data was collected using Licor Odyssey CLx software<br>Proteomic data was collected using orbitrap Fusion Lumos for SILAC samples and orbitrap Q-E Exactive for LFQ samples
Data analysis	Flow cytometry data was analyzed using FlowJo (V10) software<br>Confocal microscopy data was analyzed as follows: image processing was performed in Fiji 2.0 and vesicle tracking was performed using TrackMate for Fiji 2.0; colocalization analysis was performed in ImageJ64 using the JaCop plug-in; fractional distance analysis was performed using LAS-AF or LAS X and Excel software;<br>Western blot data was analyzed using Licor Odyssey Image Studio Light software<br>Proteomic data was analyzed as follows: raw data was converted to Mascot generic files using msconvert, and database searches were performed with MASCOT. For quantitative analysis of all SILAC samples combined, raw MS data was processed using MaxQuant (v1.5.3.1) and subsequently analyzed using Perseus software (v1.5.3.1).<br>All bar graphs and dot plots were generated using GraphPad Prism 7.0.

For manuscripts utilizing custom algorithms or software that are central to the research but not yet described in published literature, software must be made available to editors/reviewers. We strongly encourage code deposition in a community repository (e.g. GitHub). See the Nature Research guidelines for submitting code & software for further information.

Data

Policy information about availability of data

All manuscripts must include a data availability statement. This statement should provide the following information, where applicable:

- Accession codes, unique identifiers, or web links for publicly available datasets
- A list of figures that have associated raw data
- A description of any restrictions on data availability

All relevant data are available from the authors upon request.

We have made our Mass Spectrometry data sets publicly available through the ProteomeXchange Consortium via the PRIDE partner repository with the accession number PXD011899 (dataset identifier 1-20181109-74057), as indicated under the subsection 'Ubiquitome analysis' in the Methods, as well as the legend corresponding to Fig. 4.

Field-specific reporting

Please select the one below that is the best fit for your research. If you are not sure, read the appropriate sections before making your selection.

☒ Life sciences ☐ Behavioural & social sciences ☐ Ecological, evolutionary & environmental sciences

For a reference copy of the document with all sections, see nature.com/documents/nr-reporting-summary-flat.pdf

Life sciences study design

All studies must disclose on these points even when the disclosure is negative.

Sample size	for each experiment, choice of sample size has been explicitly defined in the associated figure legend
Data exclusions	no data were excluded from the analyses
Replication	replicate experiments were successfully performed and included in the analyses as indicated in the corresponding legends
Randomization	No randomization was performed. All biological and biochemical experiments were carried out with appropriate internal negative and/or positive controls as indicated.
Blinding	No blinding was used. All biological and biochemical experiments were carried out with appropriate internal negative and/or positive controls as indicated. Most results were validated from different angles and/or corroborated by alternative techniques as described in the manuscript text.

Reporting for specific materials, systems and methods

We require information from authors about some types of materials, experimental systems and methods used in many studies. Here, indicate whether each material, system or method listed is relevant to your study. If you are not sure if a list item applies to your research, read the appropriate section before selecting a response.

Materials & experimental systems

n/a	Involved in the study	
☐	☒	Antibodies
☐	☒	Eukaryotic cell lines
☒	☐	Palaeontology
☒	☐	Animals and other organisms
☒	☐	Human research participants
☒	☐	Clinical data

Methods

n/a	Involved in the study	
☒	☐	ChIP-seq
☐	☒	Flow cytometry
☒	☐	MRI-based neuroimaging

Antibodies

Antibodies used	All antibodies were specified in Methods under Antibodies and fluorescent dyes section.
Validation	The validation of each primary antibody were specified in Mehtods under Antibodies and fluorescent dyes section. If needed, the relevant references were given for the validation.

Eukaryotic cell lines

Policy information about cell lines

Cell line source(s)	MelJuSo cell line was kindly provided by Prof. G. Riethmuller (LMU, Munich). HeLa and Hek293 were purchased from ATCC.
Authentication	All the cell lines were characterized using morphology analysis and surface markers expression.
Mycoplasma contamination	All the cell lines were tested negative for mycoplasma contamination. The test was performed regularly every two month.
Commonly misidentified lines (See ICLAC register)	No misidentified cell line was used for this study.

Flow Cytometry

Plots

Confirm that:

☒ The axis labels state the marker and fluorochrome used (e.g. CD4-FITC).

☒ The axis scales are clearly visible. Include numbers along axes only for bottom left plot of group (a 'group' is an analysis of identical markers).

☒ All plots are contour plots with outliers or pseudocolor plots.

☒ A numerical value for number of cells or percentage (with statistics) is provided.

Methodology

Sample preparation	Sample preparation listed in Methods
Instrument	LSR II used for data collection.
Software	flow cytometry data collected using BD FACS DIVA (V8.0.1). Flow cytometry data analyzed using FlowJ (V10).
Cell population abundance	Cell population abundance was on the order of 80%
Gating strategy	Relevant Gating strategy shown in Supplementary Fig. 1a

☒ Tick this box to confirm that a figure exemplifying the gating strategy is provided in the Supplementary Information.
